# Postmortem Collection of Gametes for the Conservation of Endangered Mammals: A Review of the Current State-of-the-Art

**DOI:** 10.3390/ani13081360

**Published:** 2023-04-15

**Authors:** Tim E. R. G. Huijsmans, Hiba Ali Hassan, Katrien Smits, Ann Van Soom

**Affiliations:** Department of Internal Medicine, Reproduction, and Population Medicine, Faculty of Veterinary Medicine, Ghent University, Salisburylaan 133, 9820 Merelbeke, Belgium

**Keywords:** gametes, postmortem, artificial reproduction techniques, conservation, genetic resource baking, oocytes, semen

## Abstract

**Simple Summary:**

Many species are threatened with extinction. A consequence of the decreasing population sizes is the loss of genetic diversity. To maintain this genetic diversity, establishing genetic resource banks can be part of the solution. These banks contain, amongst others, sperm and oocytes, which can be used to produce offspring through artificial reproduction techniques. Deceased animals can be an important source for the collection of these gametes, without having a negative effect on living animals. To transport, collect, and store gametes of wildlife correctly, gametes of domestic animals can possibly be used to examine the ideal procedures. To date, sperm has been collected after death from at least 28 wildlife species and oocytes have been collected from at least 10 wildlife species of the Equidae, Bovidae, and Felidae families. Using these postmortem collected gametes, offspring have been produced in two wildlife species, both belonging to the Bovidae. This shows that gametes recovered postmortem can indeed be a valuable resource for the conservation of endangered species.

**Abstract:**

The collection of gametes from recently deceased domestic and wildlife mammals has been well documented in the literature. Through the utilization of gametes recovered postmortem, scientists have successfully produced embryos in 10 different wildlife species, while in 2 of those, offspring have also been born. Thus, the collection of gametes from recently deceased animals represents a valuable opportunity to increase genetic resource banks, obviating the requirement for invasive procedures. Despite the development of several protocols for gamete collection, the refinement of these techniques and the establishment of species–specific protocols are still required, taking into account both the limitations and the opportunities. In the case of wildlife, the optimization of such protocols is impeded by the scarcity of available animals, many of which have a high genetic value that must be protected rather than utilized for research purposes. Therefore, optimizing protocols for wildlife species by using domestic species as a model is crucial. In this review, we focused on the current advancements in the collection, preservation, and utilization of gametes, postmortem, in selected species belonging to Equidae, Bovidae, and Felidae, both domestic and wildlife.

## 1. Introduction

The rapid increase in the number of endangered species is a matter of great concern. Data from the IUCN Red List of Threatened Species indicates that 27% of the 5969 mammal species evaluated are facing extinction [1]. The current rate of extinction is estimated to be 1000 times the normal rate of extinction [2]. The primary threats for endangered mammals are deforestation, habitat loss, human–wildlife conflicts, hunting, and global warming, all of which contribute to biodiversity loss, the decline of populations, and a loss of genetic diversity [3]. The application of artificial reproduction technologies (ARTs) can serve as a solution to protect species from extinction. In particular, ARTs can be an indispensable tool in the conservation of endangered mammals, especially in small populations. These technologies allow for the exchange of genetic material without the need for natural breeding, enabling genetic transfer both within and between captive and free-ranging populations, thus, providing a synergistic approach to both in situ and ex situ conservation [4]. The application of ARTs in wildlife conservation eliminates the requirement for transporting animals for natural mating and acclimatization to new environments, which can cause significant stress with no guarantee of reproductive success. To make this possible, biobanks have been established across the world in zoological gardens and research institutions, such as the Frozen Zoo and the EAZA biobank [5,6]. Due to the large number of endangered species, a global prioritization system for the cryopreservation of species is required to optimize the benefits of biobanks [7]. Furthermore, ARTs eliminate the need to remove an individual from the wild to increase genetic diversity in zoos. To prevent any abuse, the Nagoya protocol, a new treaty that obliges, amongst others, researchers to negotiate binding agreements between the sending and receiving countries of gametes, is in place. The downside of this protocol is that it makes the exchange of gametes more difficult [8]. In addition, there are still challenges that make the implementation of ARTs in breeding programs for most species difficult. A lack of knowledge on species–specific reproduction traits, the low availability of studies on animals, and the difficulties in the preservation of gametes, all cause the success rates of ARTs in endangered animals to be low [9,10].

The implementation of ARTs generally requires the collection of gametes, i.e., oocytes and spermatozoa. These gametes can be obtained from both living and deceased animals [11]. The collection of samples from deceased animals does not require any interventions, e.g., electroejaculation or ovum pick-up, in the living population, making it a promising approach. To obtain functional gametes from deceased animals, several factors must be considered, including the timeframe for the postmortem, whereby samples can be collected, the effect of storage techniques on the quality of the samples, and the most appropriate ART for successful fertilization. Especially in endangered species, which often live in small populations, the quality of the gametes is already impaired, making the decrease in quality postmortem even more detrimental. A recent meta-analysis showed that genetic resource banking for the preservation of vital tissues of wildlife is feasible, especially with vitrification as a financially interesting and easily applied technique [12].

The aim of this review is to highlight the current advancements in the collection, preservation, and utilization of gametes postmortem. This review will focus on selected species from the families of Equidae, Bovidae, and Felidae in both domestic and wildlife animals and will assess the feasibility of using domestic species as a model for their wildlife counterparts. We hypothesize that collection and freezing techniques will most probably be applicable in species that are closely related to domestic animals, where backcrossing is possible and will result in viable, if not fertile, offspring. Papers reporting on the birth of hybrid offspring between horses, cattle, and domestic cats and their wildlife counterparts are summarized in Table 1. The wildlife species listed in this table are, therefore, interesting candidate species for gamete collection and preservation.

## 2. Methods

A literature search was conducted using the PubMed database, focusing exclusively on studies that addressed the use of postmortem collected sperm and oocytes from species within the families of Equidae, Bovidae, and Felidae. The search was not limited by the year of publication and all relevant papers were included up to February 2023. A broad combination of search terms related to wildlife ARTs and the collection of gametes postmortem was used. The search yielded a total of 23 relevant articles on the postmortem collection of sperm and oocytes from wildlife animals of the Equidae, Bovidae, and Felidae families.

## 3. Male Gametes

### 3.1. Anatomy

The testes of many mammals are located in the scrotum, along with their epididymis and distal spermatic cord (scrotal mammals). Exceptions to this concept are ascrotal mammals (such as seals and dolphins) and testicond mammals (such as elephants and the elephant shrew) [27]. The orientation of the testes varies among species, with the testes in horses positioned horizontally, those in cattle positioned vertically, and those in domestic cats positioned dorsocaudally. The testis consists of convoluted seminiferous tubules and spermatozoa travel from the testis through the ductuli efferentes to the epididymis, where they are stored until ejaculation. The epididymis, which is a long tightly coiled tube, is connected across the craniolateral aspect of the testis [28]. A key difference between species is the size of the epididymis (Table 2), which plays a crucial role in determining the feasibility of various postmortem sperm collection techniques. In addition to a difference in epididymal weight, there is a variation in the length of the ductus epididymis, with the ductus ranging from 70 to 90 m in horses, and from 40 to 50 m in cattle [29,30,31,32].

### 3.2. Handling and Transport of the Testes and Epididymides

When an animal has passed away, the retrieval of the testicles with the epididymis can be performed as part of a standard castration procedure. Sperm viability in relation to the storage and transportation time of the testes and epididymis has been studied in various species, such as goats, cattle, deer, and horses, thus, enabling the definition of the best methods for transporting the material to the storage facility [38,42,43,44]. A certain decrease in sperm quality and viability with increasing storage time has been well documented in Cantabric chamois [45]. This decline can be attributed to both the metabolic depletion of the sperm and the degeneration of tissue postmortem. The degenerating tissues and/or altered body fluids might pose an (epi)genetic risk to exposed sperm and prospective offspring, although these possible effects have not been assessed in mammals yet [46]. Sperm viability can be preserved for an extended period of time in horses by cooling the male reproductive organs to lower temperatures (preferably around 4 °C). This method slows down the degeneration process and reduces sperm metabolism [44].

The testicles, along with the epididymis, are usually transported in a sterile saline solution that may be supplemented with antibiotics, such as penicillin–streptomycin, to prevent bacterial growth [47]. While a temperature range of 4 °C to 5 °C is generally recommended for the transportation of testicular tissue [47], some studies, in cattle and gazelle species, have found that transportation at 18 °C to 20 °C, although for a limited time (up to 30 h), can still be effective [48,49]. However, a recent study on the impact of transportation time on the quality of epididymal spermatozoa in rams showed that transport at ambient temperatures resulted in decreased sperm motility and viability compared to transport at refrigeration temperatures. After 72 h of preservation at 17.9 to 21.5 °C, sperm motility dropped from 75 ± 2.58% to 45.83 ± 2.01% [50]. The storage of the testicles at 5 °C for 24 h has been shown to have no significant effect on sperm quality. However, after 48 h, a significant adverse effect was visible [38,39,49,51].

### 3.3. Collection Methods and Sperm Quality

Postmortem spermatozoa can be collected in vitro from the testes or epididymis. Different techniques are used for this, which are categorized based on either the source of sperm retrieval (testes or epididymis) or the method employed (mincing or aspiration). These methods have their own specific benefits and drawbacks.

In the species under consideration in this review, the collection of sperm postmortem was primarily achieved through three distinct methodologies: epididymal mincing, testicular mincing, and epididymal retrograde flushing [47,52,53]. Both mincing techniques involve either the isolation of the tissue, i.e., the cauda of the epididymis or the testis, respectively, followed by cleaning and mincing in a prewarmed extender solution. To enable the spermatozoa to swim up into the solution, the minced tissue is incubated for a designated time period. Despite filtering, the resulting spermatozoa suspension may still contain contaminants such as epithelial cells, erythrocytes, and bacteria [47,54,55]. For the retrograde flushing procedure, a fine-gauge needle attached to a tuberculin syringe containing fluid is introduced through the vas deferens to the cauda epididymis. Then, the plunger of the syringe is pulled to create negative pressure, and the needle tip is gently moved in and out within the epididymis until a clear fluid is obtained in the syringe [56]. The downside of spermatozoa collected postmortem compared to those collected at ejaculation is the lack of seminal fluid. Seminal fluid plays a species-specific role and might have a role in fertilization in a few species, while it provides some protection during freezing as well [57].

Both methods of epididymal sperm collection have been described in Equidae, Bovidae, and Felidae families, although retrograde flushing is generally preferred for horses and cattle due to the relative ease of catheterization of the ductus [58,59,60]. Therefore, the choice of sperm retrieval technique mainly depends on the species.

#### 3.3.1. Equidae

The retrograde flushing technique is considered a standard technique for the collection of epididymal spermatozoa in the Equids family. This method is characterized by its high efficiency and minimal contamination with blood [34]. The average epididymal sperm concentration after retrograde flushing in horses has been reported to be 6.5 ± 0.4 × 10^9^ spermatozoa/mL [61]. Sperm has been collected postmortem in one wildlife species, the plains zebra (*Equus quagga*), resulting in successful heterologous embryo production after ICSI with frozen-thawed epididymal semen [62,63]. Live horse foals have been born after the insemination of epididymal sperm collected post-castration via retrograde flushing [64,65]. These findings indicate the potential of semen collected postmortem from valuable wildlife for ART and genome resource banking.

#### 3.3.2. Bovidae

In the Bovidae family, the postmortem collection of sperm has been conducted in 21 wildlife species. Epididymal mincing is the most commonly used method, producing a mean sperm concentration of 3.85 × 10^10^ spermatozoa/mL in cattle [66]. Despite being used less frequently, retrograde flushing is considered the preferred method for cattle semen collection [58]. Results from artificial insemination in cattle indicate comparable pregnancy rates for frozen-thawed epididymal sperm collected via retrograde flushing (63.6%) and ejaculated semen (60.0%) [67].

#### 3.3.3. Felidae

Testicular and epididymal spermatozoa collection has been performed postmortem by testicular and epididymal mincing in six wildlife species (Table 3). The collection of epididymal sperm through epididymal mincing has a similar fertilization capacity in vitro compared to semen collected via urethral catheterization in living animals, making epididymal spermatozoa collected postmortem a valuable genetic resource for conservation [68]. The average epididymal sperm concentration retrieved by squeezing cauda and vasa deferentia was found to be 148.9 ± 102.8 × 10^6^ spermatozoa/mL in domestic cats [69], 2930 × 10^6^ spermatozoa/mL in lions [70], and 5.7 ± 1.1 × 10^6^ spermatozoa/mL in jaguars [71].

## 4. Female Gametes

### 4.1. Anatomy of the Ovaries

The ovaries are paired female gonads that play a crucial role in the development and maturation of oocytes, also known as germ cells. These organs possess both exocrine and endocrine functions and are responsible for nurturing and preparing the oocytes for ovulation [86,87]. The morphology of the ovaries varies among species, with a bean shape in horses, an oval-to-bean shape in cattle, and an oval shape in domestic cats. Ovaries are composed of the cortex and medulla. The cortex is the outer layer of the ovary and holds all the follicles, while the medulla is the inner layer and contains nerves and blood vessels. In horses, this anatomical arrangement is reversed, with the cortical tissue located exclusively on the outside of the ovulatory fossa, where ovulation occurs. The ovaries are encased by the bursa ovarica, which is formed by the mesovarium and mesosalpinx. In cattle, the ovaries are positioned along the cranial border of the broad ligament and lie parallel to the uterine horns and cervix over the pelvic floor, whereas in older cattle and during pregnancy, they are found in the abdomen. The right ovary is typically larger and more active in cattle, whereas in horses and domestic cats, the ovaries are located near the caudal region of the kidneys, with the left ovary being slightly larger in domestic cats [88,89].

### 4.2. Collection Method

It is recommended to store ovaries in a saline solution containing antibiotics during their transport to the laboratory [90]. If cat oocyte collection occurs within six hours, the ovaries can be stored at room temperature without affecting the developmental competence of the oocytes [91]. Oocytes can be harvested from the ovaries using various techniques, such as aspiration, cutting, or sieving. During aspiration, a needle is used to puncture the follicle, and the follicular fluid is drawn out [92]. The cutting method involves using a scalpel blade to make cuts over the entire surface of the ovary. The follicular wall is sometimes scraped using a curette under a stereomicroscope, causing the oocytes to fall into the culture medium for further processing [81]. The sieving method involves removing the medulla from the ovaries followed by pressing the cortices through a cell dissociation sieve. The cell solution is then passed through a series of sieves with decreasing mesh sizes, and the oocytes are recovered [84]. For routine IVF in domestic animals, ovaries are mostly collected after slaughter in cattle and horses, and after gonadectomy in cats.

Postmortem oocyte collection has been conducted using three methods, with the cutting method being preferred for horses and aspiration for cattle [93,94]. Meanwhile, both cutting and sieving methods have been used for oocyte collection in Felidae [84,95]. Therefore, the choice of method is species-dependent.

#### 4.2.1. Equidae

So far, only domestic species of Equidae have had their oocytes collected. A study in 1997 compared the different collection methods in horses and showed that the aspiration method had a low success rate, with only 31.2% recovery, compared to the cutting method with scraping, which had a higher recovery rate of 71.1% [93]. A more recent study showed a recovery rate after aspiration of 65.5% to 66.5% depending on the needle caliber [96]. As a result, cutting is preferred, where the follicle is opened with a scalpel blade, and the granulosa layer is scraped with a curette [93,97,98]. Additionally, equine oocytes can be stored overnight in a holding medium at room temperature without affecting developmental competence [99]. A study comparing the storage of oocytes in embryo holding medium at 4 °C and room temperature found higher rates of in vitro maturation, cleavage, and blastocyst formation at room temperature [100].

#### 4.2.2. Bovidae

The collection of oocytes has been conducted in three wildlife species from the Bovidae family. In cattle, the aspiration method is used to collect oocytes from the follicle and the postmortem collection of oocytes from slaughterhouse oocytes is routinely performed in bovine IVF research, yielding blastocyst rates of 30–40%. A comparison between calves and adult cattle showed that, in the case of smaller follicles, adult cattle have a higher percentage of morulae and blastocysts at day 9 after fertilization compared to calves. However, for large follicles, there is no significant difference between calves and adult cattle [94]. In terms of cleavage rate, larger follicles lead to higher rates of morulae and blastocysts in both calves and adult cattle. Our group also demonstrated that after aspiration, holding oocytes in the medium for both 6 and 10 h at room temperature did not affect the maturation rates (83.2 ± 2.9% and 78.9 ± 3.2%, respectively) nor day 8 blastocyst rates [101]. The presence of dominant follicles and corpus luteum on the ovary will also decrease the developmental quality of the collected oocytes [102], although this finding is less important in deceased animals of high genetic value, since in this case all the collected oocytes are valuable, regardless of the state of the ovary.

In sheep, the proportion of high-quality oocytes is higher in large and medium-sized oocytes compared to small oocytes [92]. These findings suggest that the size of the follicles can play a crucial role in the collection of oocytes in various species and highlight the potential value of collecting gametes from juvenile animals postmortem.

#### 4.2.3. Felidae

Oocytes have been collected in seven wildlife felid species postmortem (Table 4). The cycle stage has a noticeable effect on the developmental competence of oocytes after in vitro maturation (IVM) or in vitro fertilization (IVF). In a study of ovariohysterectomized domestic cats, the percentage of embryos that developed into blastocysts was 38.1% for females with inactive ovaries, 20.6% for females with ovaries in the follicular phase, and 36.3% for females with ovaries in the luteal phase [103]. This influence on developmental competence is likely to apply to postmortem collected oocytes as well, depending on the stage the female was in at the time of their death. This should be considered when comparing different collection methods.

## 5. Artificial Reproduction Techniques (ARTs)

### 5.1. Artificial Insemination

Artificial insemination (AI) is a commonly used ART in endangered mammals [107,108]. It offers the possibility of depositing the collected sperm into various sites of the reproductive tract, such as the vagina, cervix, or in certain cases, the uterus. Intrauterine insemination requires a lower sperm dose compared to intravaginal insemination, making the preservation of sperm in a genetic resource bank more efficient [109]. Another option is laparoscopic AI, which involves inserting sperm into the uterine lumen or oviductal ostium via laparoscopy [110]. This approach has been shown to increase pregnancy rates and conserve sperm in endangered non-domestic cat species [111]. As a result, laparoscopic AI may be a preferred option over conventional AI for sperm collected postmortem. AI has been employed in feline, bovine, and equine species, and the success rates of achieving pregnancy have varied between these species. The average pregnancy rates per cycle after AI with frozen-thawed sperm in horses are 30% to 40% [112]. Intrauterine AI in cattle results in an average conception rate of 54.7% [113]. A slightly higher conception rate, around 57%, has been achieved in domestic cats after intrauterine insemination with frozen-thawed sperm [114].

### 5.2. In Vitro Embryo Production

The procedure involves the collection of spermatozoa and oocytes, IVM of the oocytes, IVF, or intracytoplasmic sperm injection (ICSI), followed by in vitro culture (IVC) of the embryos, followed by embryo transfer to a synchronized recipient or cryopreservation of the resulting embryo until further use [115,116,117].

Although only a small number of oocytes present in the ovaries at birth are eventually ovulated over the lifetime of a female mammal, immature oocytes can be collected and subjected to IVM. Immature oocytes collected postmortem can still represent a valuable additional source of genetic material. In horses, the blastocyst rates using immature oocytes vary between 35% and 44% [118]. The blastocyst rate using immature cattle oocytes is 37.4 ± 3.3% [119]. The lowest blastocyst rate using immature oocytes, 10.7%, was obtained in the domestic cat [120]. Other research showed that the blastocyst rates in domestic cats for in vitro matured oocytes after ICSI (19%) and IVF (42%) were lower compared to in vivo matured oocytes after ICSI (30%) and IVF (48%) [121]. While ICSI achieved a success rate of 33% using in vitro matured oocytes in equine, it recorded a higher success rate of 41% from in vivo matured oocytes [122].

ICSI is a routinely used artificial reproduction technique in horses [123]. IVF was successful in horses for the first time in 1990, although it was not efficient for a long time [124]. Only recently, a repeatable standard procedure for IVF has been established [125]. In horses, successful IVF has been achieved with a sperm concentration of 1 × 10^6^ spermatozoa/mL [125], while for IVF in cattle, the sperm concentration ranges from 0.5 × 10^6^ to 2 × 10^6^ spermatozoa/mL [126]. In domestic cats, a sperm concentration of 1.5 × 10^6^ to 2.5 × 10^6^ spermatozoa/mL is typically utilized [127]. ICSI offers the advantage of direct injection of a single spermatozoon into the cytoplasm of a mature oocyte using micromanipulation, thereby eliminating the requirement for high-quality and high-quantity sperm [128,129]. This feature also enables the use of sperm from dead animals [130]. In horses, ICSI has shown greater success rates compared to IVF, and is, therefore, the preferred method [131]. The blastocyst rates after the in vitro embryo production (IVP) in horses following ovum pick up and ICSI were 19.0 ± 1.4% [132]. The blastocyst rates after ICSI in cattle were 37% and 26% for young (30 to 50 months) and aged (>120 months) cattle, respectively, compared to 67% and 50% after IVF in the same age groups, respectively [133]. In domestic cats, blastocyst rates after ICSI and IVF using in vivo matured oocytes were 43% and 53%, respectively [134]. Epididymal sperm with ICSI has resulted in a blastocyst rate of 21.9% in domestic cats [135].

An overview of the use of postmortem collected sperm and oocytes to achieve fertilization is displayed in Table 5.

## 6. Conclusions

The collection of gametes postmortem, including sperm and oocytes, has been demonstrated to be feasible in several domestic and wildlife species in the Equidae, Bovidae, and Felidae families. For the Equidae family, sperm have been collected postmortem in one wildlife species, the plains zebra. In terms of postmortem oocyte collection, this has only been accomplished in the domestic horse. In the Bovidae family, successful postmortem sperm collection has been reported in 21 wildlife species, while postmortem oocyte collection has been achieved in 3 wildlife species, with the production of offspring being reported in 2 species: the American and European bison. In the Felidae family, successful postmortem sperm collection has been reported in six species, with postmortem oocyte collection having been accomplished in seven species. Further postmortem collected gametes might be preserved in biobanks across the world.

The production of embryos using at least one gamete collected postmortem has been demonstrated using techniques such as IVF, ICSI, and AI. The utilization of domestic animals as a source of knowledge to develop protocols for the collection, transportation, and storage of gametes postmortem can serve as a valuable resource in the conservation of endangered wildlife species. Although artificial reproduction techniques are not standardized procedures in most wildlife breeding programs, they have played an important role in the conservation of some species. In elephants (*Loxodonta africana* and *Elephas maximus*), giant pandas (*Ailuropoda melanoleuca*), and black-footed ferrets (*Mustela nigripes*) AI is regularly used, and it has played an important role in conservation efforts [136,137,138]. A recent study showed that the inclusion of ARTs and biobanking in the captive management of koalas (*Phascolarctos cinereus*) would be feasible and beneficial for genetic diversity [139]. For the Asiatic golden cat (*Catopuma temmincki*), ARTs are the only hope to maintain the captive European population, with only a few females left, as natural mating is almost impossible due to the overaggressive behavior of the males. An AI in this species already resulted in the birth of a healthy twin [140]. In addition, the widespread research and successes in domestic animals have shown that establishing successful protocols is possible.

As the postmortem collection procedure is noninvasive, it allows researchers to establish and optimize collection, transportation, preservation, maturation, and fertilization techniques without the involvement of any living animals. Therefore, they have no negative impact on animal welfare and carry no risk for both humans and animals. In addition, the use of postmortem collected gametes for research is more cost-effective. Unfortunately, the infield application of gametes collected postmortem is limited due to the decrease in quality of the gametes over time, which results in lower blastocyst rates compared to gametes derived from living animals [62,141].

To turn the use of postmortem collected gametes into a reproductive success, knowledge gaps in reproductive biology need to be addressed. Collection and preservation methods need to be optimized to allow us to work with good-quality gametes, which result in higher chances of fertilization, and better-quality embryos. The right timing of AI and embryo transfer is key and requires knowledge of the monitoring of the cycle or strategies to synchronize the recipient. Opportunities for the extrapolation of knowledge between species need to be investigated as the diversity in reproductive biology might jeopardize the extrapolation of data between species. By addressing these challenges, ARTs can be part of the solution to protect species from extinction, with postmortem collected gametes as part of the resources that can be used. Therefore, the collection of gametes postmortem can play a substantial role in addressing the loss of genetic diversity and preserving genetic resources.

## Figures and Tables

**Table 1:** Overview of hybrids between domestic and wildlife animals, born within the Equidae, Bovidae, and Felidae families.

Domestic Animal	Family	Wildlife Animal	Reference
Domestic horse (*Equus caballus*)	Equidae	Mountain zebra (*Equus zebra*)	[13]
		Plains zebra (*Equus quagga*)	[14]
		Grévy’s zebra (*Equus grevyi*)	[14]
		Donkey (*Equus asinus*)	[15]
		Przewalski’s horse (*Equus przewalskii*)	[16]
Domestic cattle (*Bos taurus*)	Bovidae	American bison (*Bison bison*)	[17]
		European bison (*Bison bonasus*)	[18]
		Banteng (*Bos javanicus*)	[19]
		Wild yak (*Bos mutus*)	[19]
Domestic cat (*Felis catus*)	Felidae	Serval (*Leptailurus serval*)	[20]
		Leopard cat (*Prionailurus bengalensis*)	[21]
		Sand cat (*Felis margarita*)	[22]
		Fishing cat (*Prionailurus viverrinus*)	[23]
		Scottish wildcat (*Felis silvestris*)	[24]
		Caracal (*Caracal caracal*)	[25]
		Jungle cat (*Felis chaus*)	[26]

**Table 2:** Weight of the testis and epididymis and the total sperm output for a few example species in the Equidae, Bovidae, and Felidae families.

Family	Species	Testis Weight (Grams)	Epididymal Weight (Grams)	Total Sperm Output	Reference
Equidae	Horse	103.0 ± 6.3	18.5−21.5	1.9–7.5 × 10^9^	[33]
	Donkey	-	14.2 ± 2.3	6.4–14.8 × 10^9^	[34]
	Egyptian donkey	91.92 ± 4.66	2.52 ± 0.12	-	[35]
Bovidae	Cattle	272 ± 11	23 ± 1	13.3 ± 0.7 × 10^9^	[36]
	Awassi sheep	157.6	22.5 ± 1.1	55.8 × 10^9^	[37]
	Moufflon	191.11 ± 4.9	34.36 ± 0.7	-	[38]
	Red Sokoto goat	56.3	6.97	-	[39]
	Afar goat	67.6 ± 3.49	10.7 ± 1.20	-	[40]
	Long-eared Somali goat	74.8 ± 5.81	11.1 ± 1.44	-	[40]
	Woyto-Guji goat	67.6 ± 3.97	7.92 ± 1.06	-	[40]
Felidae	Domestic cat	1.49 ± 0.52	0.31 ± 0.06	-	[41]

**Table 3:** Overview of the Equidae, Bovidae, and Felidae species, in which sperm have been collected postmortem and the method used.

Family	Species	Technique	Transportation Temperature	Semen ConcentrationmL^−1^	Motility	Production of Embryo or Offspring	Reference
Equidae	Plains zebra (*Equus quagga*)	No data	No data	No data	No data	+	[63]
		Retrograde flushing	No data	No data	No data	+	[62]
Bovidae	Spanish ibex (*Capra pyrenaica*)	Epididymal mincing	12 °C	No data	87.1%	-	[72]
		Epididymal mincing	12 °C	No data	78.1%	+	[73]
		Retrograde flushing	9–11 °C	No data	83.7%	+	[74]
	Sumatran serows (*Capricorns sumatraensis sumatraensis*)	Testicular mincing	4–5 °C	No data	No data	-	[47]
	Cantabric chamois (*Rupicapra pyrenaica parva)*	Epididymal mincing	4–5 °C	3.8 × 10^9^	85.0%	-	[45]
		Epididymal mincing	Refrigerated	No data	68.7%	-	[75]
	Mountain gazelle (*Gazella gazella*)	Epididymal mincing	Ambient temperature	No data	60–80%	-	[48]
	Dorcas gazelle (*Gazella dorcas*)	Epididymal mincing	Ambient temperature	No data	75%	-	[48]
	European bison (*Bison bonasus*)	Epididymal mincing	15 °C	3.2 × 10^7^	60–90%	+	[76]
		Epididymal mincing	No data	1.90 × 10^9^	No data	-	[77]
		Epididymal mincing	No data	No data	No data	+	[78]
	Moufflon (*Ovis musimon*)	Epididymal mincing	22 °C	3.7 × 10^9^	80.3%	+	[38]
		Retrograde flushing	No data	No data	61.3%	-	[79]
		Retrograde flushing	4 °C	No data	No data	-	[53]
	Barbary sheep (*Ammotragus lervia*)	Retrograde flushing	4 °C	No data	No data	-	[53]
	Springbok (*Antidorcas marsupialis*)	Retrograde flushing	4–5 °C	No data	86.2%	-	[80]
		Retrograde flushing	4 °C	No data	No data	-	[53]
	Gemsbok (*Oryx gazella*)	Retrograde flushing	4 °C	No data	No data	-	[53]
	Southern lechwe (*Kobus leche*)	Retrograde flushing	4 °C	No data	No data	-	[53]
	Defassa waterbuck (*Kobus ellipsiprymnus*)	Retrograde flushing	4 °C	No data	No data	-	[53]
	Impala (*Aepyceros melampus*)	Retrograde flushing	4–5 °C	No data	82.9%	-	[80]
		Retrograde flushing	4 °C	No data	No data	-	[53]
	Blesbok (*Damaliscus pygargus phillipsi*)	Retrograde flushing	4–5 °C	No data	85.8%	-	[80]
		Retrograde flushing	4 °C	No data	No data	-	[53]
	Nilgai (*Boselaphus tragocamelus*)	Epididymal mincing	No data	No data	50–60%	+	[81]
	Blue wildebeest (*Connochaetes taurinus*)	Retrograde flushing	4 °C	No data	No data	-	[53]
	Cape eland (*Taurotragus oryx*)	Retrograde flushing	4 °C	No data	No data	-	[53]
	Greater kudu (*Tragelaphus strepsiceros*)	Retrograde flushing	4 °C	No data	No data	-	[53]
	Sitatunga (*Tragelaphus spekii*)	Retrograde flushing	4 °C	No data	No data	-	[53]
	Dwarf forest buffalo (*Syncerus caffer nanus*)	Retrograde flushing	4 °C	No data	No data	-	[53]
	Swamp buffalo (*Bubalus bubalis*)	Epididymal mincing	On ice	2.1 × 10^8^	66%	-	[82]
Felidae	Jungle cat (*Felis chaus*)	Testicular mincing	4–5 °C	No data	No data	-	[47]
	Tiger (*Panthera tigris*)	Epididymal mincing	20–24 °C	5.2 × 10^7^	38.3%	-	[83]
	Lion (*Panthera leo*)	Testicular mincing	4–5 °C	No data	No data	-	[47]
		Epididymal mincing	No data	No data	No data	+	[84]
	Leopard (*Panthera* *pardus*)	Testicular mincing	4–5 °C	No data	No data	-	[47]
		Epididymal mincing	No data	No data	No data	+	[84]
	Puma (*Felis concolor*)	Epididymal mincing	No data	No data	No data	+	[84]
		Epididymal mincing	4 °C	No data	No data	-	[85]
	Jaguar (*Panthera onca*)	Epididymal mincing	No data	No data	No data	+	[84]

**Table 4:** Overview of the Bovidae and Felidae species, in which oocytes have been collected postmortem and the collection method.

Family	Species	Technique	Transportation Temperature	Number of Oocytes	% IVM	Production of Embryo or Offspring	Reference
Bovidae	American Bison (*Bison bison*)	Aspiration	25–28 °C	9836	No data	+	[104]
	European bison (*Bison bonansus*)	Aspiration	30 °C	50	84.3% *	+	[78]
	Nilgai (*Boselaphus tragocamelus*)	Cutting	38.5 °C	517	63.6%	+	[81]
Felidae	Lion (*Panthera leo*)	Sieving	No data	66	69.7%	-	[84]
		Cutting	Immediately processed	119 **	52.1% **	+	[105]
		Cutting	37 °C	33	53.8%	-	[95]
	Tiger (*Panthera tigris*)	Sieving	No data	63	54.0%	-	[84]
		Cutting	37 °C	22	56.3%	-	[95]
	Leopard (*Panthera* *pardus*)	Sieving	No data	9	55.6%	-	[84]
		Cutting	37 °C	64	58.7%	-	[95]
	Eurasian lynx (*Lynx lynx*)	Cutting	4 °C	47	48.0% *	-	[106]
	Serval (*Leptailurus* *serval*)	Cutting	4 °C	30	No data	-	[106]
	Pallas’s cat (*Felis manul*)	Cutting	4 °C	104	50.0% *	-	[106]
	Puma (*Felis concolor*)	Sieving	No data	25	80.0%	-	[84]

*: %IVM of for maturation selected oocytes; **: partly fresh, partly vitrified.

**Table 5:** Overview of the Equidae, Bovidae, and Felidae species, in which postmortem sperm and/or oocytes have been used as gametes in an artificial reproduction technique, including whether offspring has been produced or not.

Family	Species	PM Gamete	Technique	Results	Reference
Equidae	Plains zebra (*Equus quagga*)	Sperm	ICSI with horse oocyte	6.12%, day 11 blastocyst rate No embryo transfer	[63]
		Sperm	ICSI with horse oocyte	7% blastocyst rate No embryo transfer	[62]
Bovidae	European bison (*Bison bonasus*)	Sperm	AI	2/30 (6.7%) gave birth to hybrid calves	[76]
		Sperm and Oocyte	IVF	10.7% morula plus early blastocyst rate 3/5 (60%) pregnant after interspecies embryo transfer No offspring	[78]
	American bison (*Bison bison*)	Oocyte	IVF	8.3% blastocyst rate 1/10 (10%) gave birth	[104]
	Nilgai (*Boselaphus tragocamelus*)	Sperm and Oocyte	IVF	42% cleaved 0% blastocyst rate	[81]
	Spanish ibex (*Capra pyrenaica*)	Sperm	AI	1/6 (16.7%) gave birth	[73]
		Sperm and Oocyte	IVF with domestic goat oocyte	21.2% blastocyst rate No embryo transfer	[74]
	Moufflon (*Ovis musimon*)	Sperm	IVF	53% cleaved No embryo transfer	[38]
Felidae	Lion (*Panthera leo*)	Sperm and Oocyte	IVF of lion oocyte with lion semen + domestic cat semen	31.6% embryos > 8 cells with lion semen 0% embryos > 8 cells with domestic cat semen No embryo transfer	[84]
		Oocyte	ICSI	24.1% cleaved 0% blastocyst rate	[105]
	Tiger (*Panthera tigris*)	Sperm and Oocyte	IVF with domestic cat semen	0% embryos > 8 cells	[84]
	Leopard (*Panthera pardus*)	Sperm and Oocyte	IVF with domestic cat semen	22.2% embryos > 8 cells No embryo transfer	[84]
		Sperm	IVF with domestic cat oocyte	19.5% embryos > 8 cells No embryo transfer	[84]
	Puma (*Felis concolor*)	Sperm and Oocyte	IVF	20% embryos > 8 cells No embryo transfer	[84]
